# Monitoring of Antimicrobial Resistance of *Salmonella* Serotypes Isolated from Humans in Northwest Italy, 2012–2021

**DOI:** 10.3390/pathogens12010089

**Published:** 2023-01-05

**Authors:** Monica Pitti, Aitor Garcia-Vozmediano, Clara Tramuta, CeRTiS Clinical Laboratories Group, Cristiana Maurella, Lucia Decastelli

**Affiliations:** 1 Centro di Riferimento per la Tipizzazione delle Salmonelle, CeRTiS, Istituto Zooprofilattico Sperimentale del Piemonte Liguria e Valle d’Aosta, Via Bologna, 148, 10154 Turin, Italy; 2 S.S. Rischi Alimentari ed Epidemiologia degli Alimenti (REA), Istituto Zooprofilattico Sperimentale del Piemonte Liguria e Valle d’Aosta, Via Bologna, 148, 10154 Turin, Italy

**Keywords:** foodborne infections, drug-resistant bacteria, *Salmonella* spp., disease surveillance

## Abstract

*Salmonella enterica* is among the most common causes of foodborne outbreaks in humans in Europe. The global emergence of resistance to antimicrobials calls for close monitoring of the spread and prevalence of resistant *Salmonella* strains. In this study, we investigated the occurrence of antimicrobial resistance of *Salmonella* serotypes isolated from humans between 2012 and 2021 in Piedmont, northwest Italy. A total of 4814 *Salmonella* strains (168 serotypes) were tested against six classes of antimicrobials. Many strains (83.3%) showed resistance to at least one antibiotic: tetracycline (85.1%), ampicillin (79.2%), quinolones (47.4%), and gentamicin (28.4%). Between the first (2012–2016) and the second study period (2017–2021), a decrease in antimicrobial resistance was noted for tetracycline (from 92.4% to 75.3%), ampicillin (from 85.3% to 71.3%), quinolones (from 49.4% to 44.6%), and cefotaxime (from 34.8% to 4.0%). Many multidrug resistant *Salmonella* strains (43.6%) belonged to *S.* ser. Typhimurium, *S.* ser. Infantis, and *S.* ser. Typhimurium 1,4,[5],12:i:-. Overall, multidrug resistance decreased from 60.7% to 26.4%, indicating a reduction in the antimicrobial resistance of *Salmonella* strains in Piedmont and in Europe and demonstrating the effectiveness of the measures that were put in place to reduce antimicrobial resistance.

## 1. Introduction

*Salmonella enterica* is the leading cause of foodborne outbreaks in the European Union (EU). In 2020 alone, 94 foodborne outbreaks of salmonellosis were reported, causing 3686 cases of illness, 812 hospitalizations, and seven deaths [1]. In 2021, 60,494 laboratory-confirmed cases of non-typhoidal salmonellosis were reported in the EU, with an increase of 14% in cases, compared to 2020. Seventy-three cases were fatal, accounting for a case fatality rate of 0.19%. The EU notification rate for salmonellosis was 16.6 cases per 100,000 population. Italy reported 3768 cases with a notification rate of 6.4 per 100,000 population [2].

*Salmonella* is a ubiquitous bacterium of which six subspecies and more than 2600 serotypes are known. Serotypes of the species *Salmonella enterica* can be classified as typhoid and nontyphoid (NTS), based on differences in host specificity, virulence, and severity of the clinical manifestations they cause in humans [3]. Typhoid *Salmonella* strains include *S.* ser. Typhi and *S.* ser. Paratyphi, which are human host-restricted organisms that cause enteric fever, a severe systemic syndrome with moderate to high fatality rates when untreated [4,5]. By contrast, NTS strains usually cause self-limiting gastrointestinal infections in humans. They are often acquired through the consumption of contaminated animal food products made from domestic and wild animals, which are the natural reservoirs. Furthermore, fruits and vegetables can also serve as vehicles for pathogen transmission [6,7]. In Europe, pig and poultry food products are associated with nontyphoid human infections, most often caused by *S.* ser. Enteritidis, followed by *S.* ser. Typhimurium, *S.* ser. Typhimurium 1,4,[5],12:i:-, *S.* ser. Infantis, and *S.* ser. Derby [1]. Because NTS strains cause self-limiting gastrointestinal infections in humans, antimicrobial treatment is not normally required. In a small percentage of cases, however, the infection is invasive (iNTS) and antimicrobial treatment is essential and life-saving [8]. In sub-Saharan Africa iNTS infections particularly affect immunosuppressed populations, with children (<5 years) experiencing a mortality rate of 20–30%. *Salmonella* ser. Typhimurium (77.4%), *Salmonella* ser. Enteritidis (17.0%), and *Salmonella* ser. Dublin (0.1%) have been recorded as the main serovars involved in iNTS infections during the period from 1996 to 2016 [9]. Similarly, *S*. ser. Typhimurium was the most common serovar in iNTS infections from Nigeria, registering a prevalence of 39.8% during 1999–2018 and followed by *S.* ser. Enteritidis (29.3%) [10]. Infants, young children, the elderly, and the immunocompromised are at particular risk for bacteremia, in which multi-resistant strains are also more likely to cause invasive disease [11]. 

Multidrug resistant (MDR) *Salmonella* strains are defined as resistant to three or more antimicrobial classes [12]. The increasing resistance against first-line antimicrobials (aminopenicillins, trimethoprim-sulfamethoxazole, chloramphenicol) in the treatment of salmonellosis has led to a shift in treatment to fluoroquinolones and third-generation cephalosporins [8] and resistance has emerged with the wider use of fluoroquinolones over the last twenty years [11]. 

Moreover, some *Salmonella* serotypes have developed resistance towards broad-spectrum cephalosporins through mutations in genes encoding extended-spectrum β-lactamases [13]. The global emergence of resistance to antimicrobials calls for close monitoring of the spread and prevalence of resistant and multi-resistant strains and to detect possible qualitative and quantitative variations over time. 

Our institute (Istituto Zooprofilattico Sperimentale del Piemonte, Liguria e Valle d’Aosta, IZSPLV) supports public health agencies in human/animal outbreak investigations in northwest Italy, in addition to carrying out diagnostic testing and research activities in national surveillance programs for animal health and food safety. In 2011, IZSPLV was designated a regional reference center for *Salmonella* typing (Centro di Riferimento per la Tipizzazione delle Salmonelle, CeRTiS) within the national surveillance networks for enteric pathogens in human medicine (Enter-NET). CeRTiS is involved in the identification and characterization of enteric pathogens. The institute performs serotyping and investigates the antimicrobial resistance (AMR) profiles of *Salmonella* strains. Surveillance data on enteric pathogens generated by CeRTiS activities are regularly sent to the Italian National Institute of Health (ISS) and then to the European Centre for Disease Prevention and Control within the frame of the European Food- and Waterborne Diseases and Zoonoses Network (FWD-Net). 

The aim of the present study was to investigate the prevalence of *Salmonella* serotypes and the occurrence of antimicrobial resistance in strains isolated from humans in Piedmont between 2012 and 2021.

## 2. Materials and Methods

### 2.1. Human Sample Collection and Salmonella Serotyping

CeRTiS receives samples of enteric pathogens isolated from symptomatic human patients presenting at health care centers. A total of 22 clinical laboratories located in Piedmont (northwest Italy) sent CeRTiS *Salmonella* strains isolated from cases of human infections. These *Salmonella* strains are previously identified using the Vitek^®^2 System (Biomerieux, Marcy l’Étoile, France) or Wellcolex Color Salmonella Test (ThermoFisher Scientific, Waltham, MA, USA) following the manufacturers’ instructions. A total of 4814 *S. enterica* strains isolated from 2012 to 2021 were analyzed. The isolates were obtained from samples of feces (*n* = 4518), urine (*n* = 111), blood (*n* = 129), purulent exudate (*n* = 6), cerebrospinal fluid (*n* = 1), and other biological fluids (*n* = 49). The strains were subcultured on Columbia Blood Agar (Becton&Dickinson, Franklin Lakes, NJ, USA) at 37 °C for 24 h and then serotyped according to the Kaufmann-White and Le Minor scheme [14] using O and H antisera (Statens Serum Institut, Artillerivej, Denmark). 

### 2.2. Antimicrobial Susceptibility Testing

*Salmonella* strains were tested for antimicrobial susceptibility using the agar disk diffusion method, according to the European Committee on Antimicrobial Susceptibility Testing (EUCAST) guidelines [15]. The antimicrobial panels included the following antibiotics and disk contentBiolab ZRT, Hungary): ampicillin 10 μg (AMP), cefotaxime 5 μg (FOT), chloramphenicol 30 μg (CHL), ciprofloxacin 5 μg (CIP), gentamicin 10 μg (GEN), nalidixic acid 30 μg, (NAL), and tetracycline 30 μg (TET). Breakpoints described by EUCAST [16] and the Clinical and Laboratory Standards Institute (CLSI) were used [17,18] and strains displaying intermediate susceptibility were considered resistant. Furthermore, strains showing concurrent resistance to at least three classes of antibiotics (aminoglycosides, cephalosporins, penicillins, phenicols, quinolones, tetracyclines) were defined as multidrug-resistant (MDR) strains. 

### 2.3. Statistical Analysis

Data analysis was performed using Stata 16 [19]. The binomial exact test was applied to calculate the prevalence of the *Salmonella* serotypes identified and to summarize the prevalence of AMR for each class of antibiotics and their combinations. We evaluated temporal patterns in prevalence and AMR of *Salmonella* strains with the non-parametric Wilcoxon-type test for trend [20]. Comparisons of infection prevalence between serotypes according to the source of the samples were made using Pearson’s chi-squared test. We also applied this test to compare AMR against single drug and MDR patterns among *Salmonella* serotypes during the first study period (2012–2016) and the second (2017–2021). Statistical significance was set at *p* < 0.05. 

## 3. Results

### 3.1. Salmonella Serotyping

**Figure 1:**
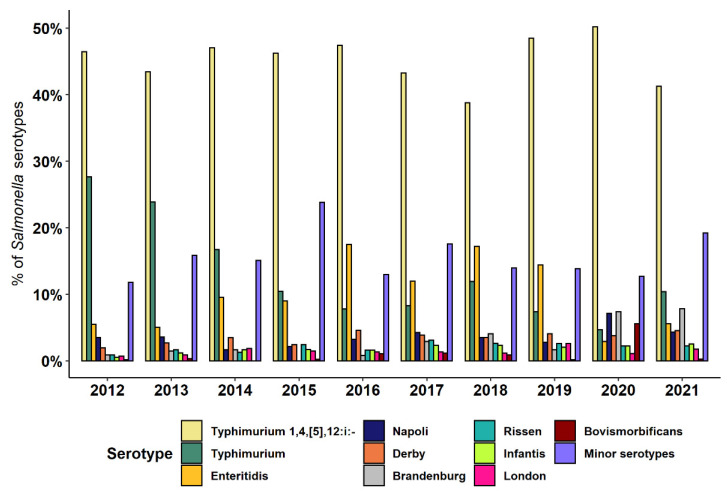
Prevalence of *Salmonella* serotypes involved in human infections in Piedmont during 2012–2021.

A total of 168 *Salmonella* serotypes were identified (Table S1), albeit few were responsible for the majority of human infection cases (Table 1). *S.* ser. Typhimurium 1,4,[5],12:i:-, *S.* ser. Typhimurium, and *S.* ser. Enteritidis were predominant during almost the entire time period, except for the last two years when *S.* ser. Brandenburg (in both 2020 and 2021) and *S.* ser. Napoli (in 2020) were the most frequent serotypes together with *S.* ser. Typhimurium 1,4,[5],12:i:- (Figure 1). The distribution of *Salmonella* serotypes differed during the study period, with a sharp decrease in the occurrence of *S.* ser. Typhimurium (Wilcoxon-type test for trend, *p* < 0.05) and a generally stable occurrence of *S.* ser. Typhimurium 1,4,[5],12:i: and *S.* ser. Enteritidis. The prevalence of the remaining seven most frequent serotypes increased over the years, especially *S.* ser. Brandenburg (prevalence, 0.9% in 2012 vs. 7.8% in 2021; *p* < 0.05), *S.* ser. Infantis (0.5% vs. 2.5%; *p* < 0.01), and *S.* ser. Derby (1.9% vs. 4.6%; *p* < 0.05), while no substantial changes in prevalence were detected for *S.* ser. Napoli (prevalence, 3.5% vs. 4.3%) during the study period (Figure 1). 

The ten most frequent serotypes were isolated from 3963 human patients, most of whom showing gastrointestinal infections (*n* = 3885). These serotypes were also recorded as causative agents of extraintestinal infections, including urinary tract infections (*n* = 88) and bacteriemia (*n* = 88). A single case of neuroinvasive infection caused by *S.* ser. Typhimurium was detected after isolating the bacterium from the cerebrospinal fluid. Serotypes *S.* ser. Derby, *S.* ser. Enteritidis, and *S.* ser. Typhimurium were also isolated from three different infections involving pus samples. *S.* ser. Typhimurium 1,4,[5],12:i:-, *S.* ser. Typhimurium, and *S.* ser. Enteritidis were the most frequently isolated in cases of bacteriemia, and showed comparable prevalence (*p* > 0.05). By contrast, *S.* ser. Typhimurium 1,4,[5],12:i:- was most often detected in urinary tract infections (*p* < 0.001), accounting for 31.8% (95% CI = 22.3–42.6) of cases, followed in frequency by *S.* ser. Derby (17.0%; 95% CI = 9.9–26.6), *S.* ser. Typhimurium (13.6%; 95% CI = 7.2–22.6), and *S.* ser. Enteritidis (6.8%; 95% CI = 2.5–14.3).

### 3.2. Antimicrobial Resistance 

**Figure 2:**
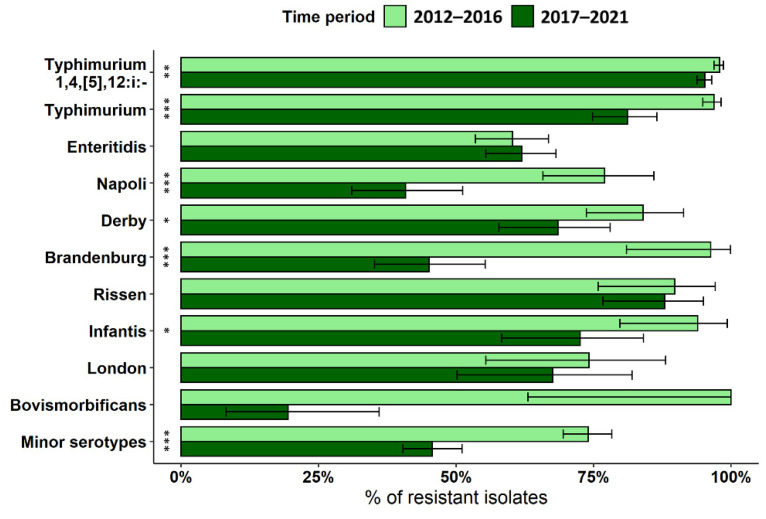
Prevalence and 95% CI of AMR in the most common serotypes recovered in the two halves of the study period. Prevalence is expressed as the number of isolates that showed resistant phenotypes against at least one drug. Asterisks denote significant differences in AMR between the two time periods (Pearson’s chi-squared test, * *p* < 0.05, ** *p* < 0.01, *** *p* < 0.001).

Antimicrobial resistance (AMR) was generally common in the *Salmonella* isolates investigated, with 83.3% (95% CI = 82.2–84.4) of the strains displaying resistance to at least one drug. However, antimicrobial susceptibility differed between the three most frequent (13.5%; 95% CI = 12.4–14.5) and minor *Salmonella* serotypes (34.4%; 95% CI = 31.0–38.0; Pearson’s chi-squared test, *p* < 0.001). The prevalence of antimicrobial resistant *Salmonella* strains decreased during the study period (Wilcoxon-type test, *p* < 0.05), with an annual prevalence of 94.4% (95% CI = 92.1–96.1) in 2012 and 64.6% (95% CI = 59.8–69.4) in 2021. This trend was evident for the ten most frequent serotypes between the first and the second half of the study period, except for *S.* ser. Enteritidis, *S.* ser. Rissen, and *S.* ser. London in which resistance levels remained unchanged over the same period (Figure 2). However, the pattern for *S*. ser. Bovismorbificans was difficult to assess because the sample size varied considerably (8/8 resistant isolates in 2012–2016 vs. 7/37 in 2017–2021). 

We generally observed differences in AMR according to the type of infections (*p* < 0.001). *Salmonella* strains involved in gastrointestinal infections experienced the highest levels of AMR (80.7%; 95% CI = 79.5–81.9) compared with those involved in urinary tract infections (73.9%; 95% CI = 64.7–81.8) and bacteriemia (68.2%; 95% CI = 59.4–76.1), which displayed comparable resistance levels (*p* > 0.05). 

**Figure 3:**
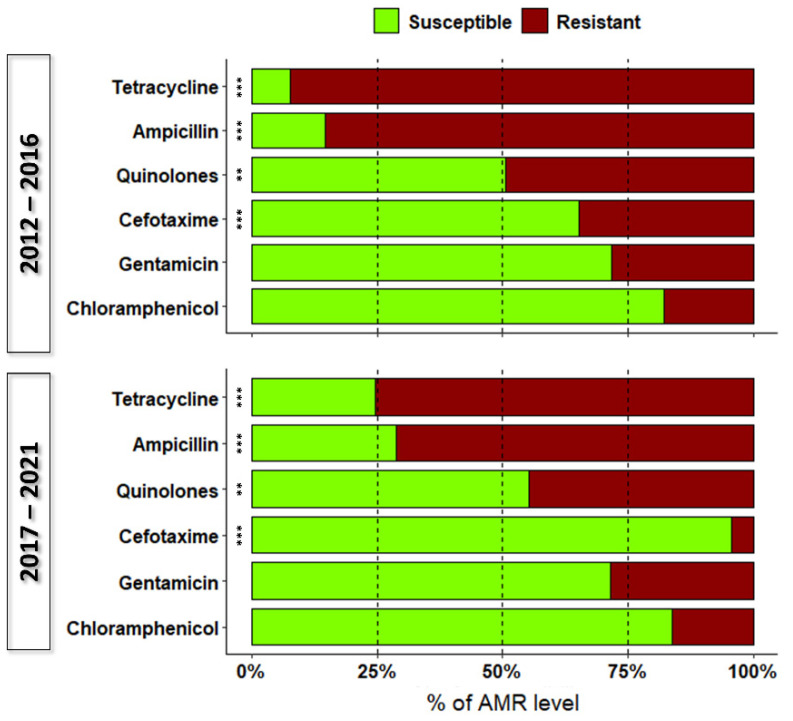
Percentage of susceptibility and resistance of *Salmonella* strains causing infections in humans in the two halves of the study period. Asterisks denote significant differences in AMR against each antibiotic between the two time periods (Pearson’s chi-squared test, ** *p* < 0.01; *** *p* < 0.001).

Only 17.9% (95% CI = 16.7–19.2) of *Salmonella* strains were resistant to a single antibiotic class. The highest levels of AMR were observed against tetracycline and ampicillin, with 85.1% (95% CI = 83.9–86.3) and 79.2% (95% CI = 77.8–80.6) of *Salmonella* strains exhibiting resistance against these antimicrobials, respectively. These antibiotics were followed by the quinolones, nalidixic acid, and ciprofloxacin, with a joint AMR prevalence of 47.4% (95% CI = 45.7–49.1), gentamicin (28.4%; 95% CI = 26.8–29.9), and cefotaxime (21.2%; 95% CI = 19.9–22.6). The lowest resistance was displayed against chloramphenicol (17.1%; 95% CI = 15.8–18.4). Antimicrobial resistance differed for most drugs tested between the two time periods (Pearson’s chi-squared test, *p* < 0.001; Figure 3), except for gentamicin and chloramphenicol for which the level of AMR remained constant (*p* > 0.05). Resistance against the combination of two antibiotics was particularly frequent for *S.* ser. Typhimurium 1,4,[5],12:i:-, with 45.4% (95% CI = 43.3–47.5) of strains showing this pattern. We recorded 14 different two-drug combinations; AMR against ampicillin–tetracycline (67.8%; 95% CI = 65.3–70.3) and quinolones–gentamicin (13.5%; 95% CI = 11.7–15.4) combinations was common.

**Figure 4:**
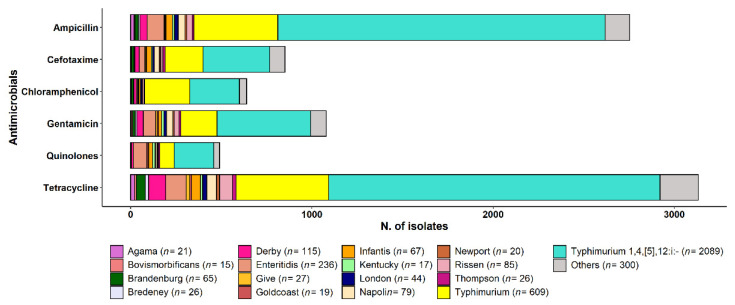
Number of strains resistant against the six classes of antimicrobials in each *Salmonella* serotype. Serotypes with fewer than 10 strains are summed and denoted as “Others”.

Antimicrobial resistance to tetracycline (TET) and ampicillin (AMP) was extremely high in *S.* ser. Agama (TET 100%; AMP 95.2%), *S.* ser. Typhimurium 1,4,[5],12:i:- (TET 87.6%; AMP 86.5%), *S.* ser. Newport (TET 85%; AMP 55%), *S.* ser. Typhimurium (TET 83.9%; AMP 76%), *S.* ser. Rissen (TET 83.5%; AMP 34.1%), and *S.* ser. Derby (TET 81.7%; AMP 33.9%) (Figure 4).

#### Multidrug Resistance (MDR)

Multidrug resistance patterns (≥3 classes of antibiotics) were rather frequent, accounting for 43.6% (95% CI 42.0–45.3) of the resistant strains. We observed the highest levels of MDR in *S.* ser. Typhimurium, *S.* ser. Infantis, and *S.* ser. Typhimurium 1,4,[5],12:i:-, which accounted for 63.3%, 52.9%, and 44.7% of resistant strains, respectively, followed in frequency by *S.* ser. Napoli (36.1%), *S.* ser. Derby (27.4%), *S.* ser. London (27.1%), S. ser. Rissen (25.6%), *S.* ser. Brandenburg (20.8%), and *S.* ser. Enteritidis (14.8%). 

**Figure 5:**
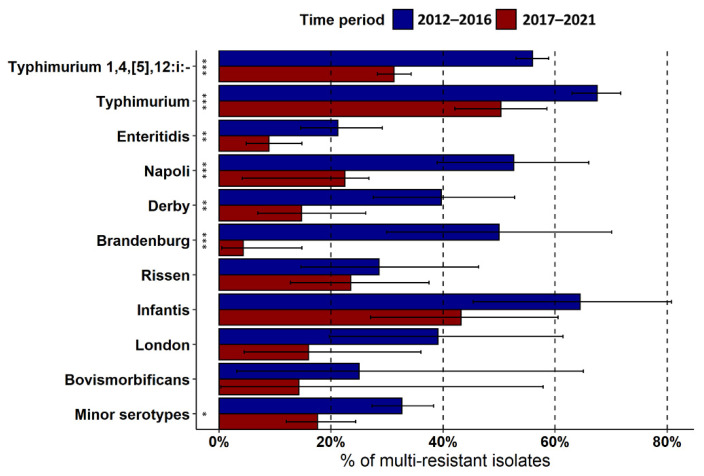
Prevalence and 95% CI of MDR in the most frequent serotypes recorded in the two halves of the study period. Asterisks denote significant differences in the prevalence of MDR between the two study periods (Pearson’s chi-squared test, * *p* < 0.05; ** *p* < 0.01; *** *p* < 0.001).

The overall prevalence of MDR in the prevailing serotypes decreased significantly over the years (Pearson’s chi-squared test, *p* < 0.001) and was, on average, 60.7% (95% CI = 56.2–65.1) in 2012 and 26.4% (95% CI = 20.9–32.5) in 2021. This trend was plainly evident for most of the prevailing serotypes, except for *S.* ser. Rissen, *S.* ser. Infantis, and *S.* ser. London (Figure 5). With regards to the type of infections, we detected comparable levels of MDR between strains involved in gastrointestinal (34.7; 95% CI = 33.2–36.3), urinary infections (34.1%; 95% CI = 24.0–45.4), and cases of bacteriemia (27.3; 95% CI = 18.3–37.8).

Multidrug resistance patterns against the six antimicrobials classes tested (AMP–FOT–CHL–GEN–TET–CIP/NAL) was observed in 87 strains. Six different combinations of antimicrobials were observed in MDR phenotypes involving five antibiotics with AMP–FOT–GEN–TET–CIP/NAL (53.5%; 95% CI 47.4–59.5), AMP–CHL–GEN–TET–CIP/NAL (22.9%; 95% CI = 18.1–28.3), and AMP–FOT–CHL–TET–CIP/NAL (14.9%; 95% CI = 10.9–19.7) being the most prevalent and observed in 275 strains. Higher numbers of combinations were ascertained in MDR phenotypes involving three (*n* = 18) and four (*n* = 13) drugs. These latter occurred in 506 strains, in which the most frequent combinations were AMP–FOT–TET–CIP/NAL (29.1%; 95% CI = 25.1–33.2), AMP–GEN–TET–CIP/NAL (28.3%; 95% CI = 24.4–32.4), and AMP–CHL–TET–CIP/NAL (16.6%; 95% CI = 13.5–20.1). The combination AMP–TET–CIP/NAL (43.7%; 95% CI = 40.0–47.6) prevailed among strains displaying resistance to three antibiotics (*n* = 667), followed by AMP–CHL–TET (17.2%; 95% CI = 14.4–20.3), and AMP–FOT–TET (9.4%; 95% CI = 7.3–11.9).

## 4. Discussion

Global monitoring of the emergence of antimicrobial resistance of *Salmonella* strains is essential for protecting public health. In developing countries, for example, the spread of antimicrobial resistant *Salmonella* strains can cause more acute and invasive infections, in addition to treatment failure and greater risk of mortality [21].

Here we analyzed the AMR of *Salmonella* serotypes isolated from human biological samples between 2012 and 2021 against six antimicrobial classes and 52 antimicrobial combinations. We found highly diverse resistance pattern phenotypes. Among the 168 different *Salmonella* serotypes involved in human infections, the three most frequent were *S.* ser. Typhimurium 1,4,[5],12:i:-, *S*. Typhimurium, and *S.* ser. Enteritidis, which were detected over almost the entire study period, as previously reported by global studies [1,22,23]. In addition, 3.6% and 3.4% of the isolates were *S.* ser. Napoli and *S*. ser. Derby, respectively. The most detected serotype in Piedmont was *S.* ser. Typhimurium 1,4,[5],12:i:-, which is consistent with previous reports published at national level [24]. By contrast, *S*. ser. Enteritidis is the serovar that is more frequently identified in EU, however an increase in prevalence has been observed for *S.* ser. Typhimurium 1,4,[5],12:i:- while S. ser. Enteritidis is following a decreasing trend [1].

Among the iNTS strains, the most frequent serotypes were *S*. ser. Typhimurium 1,4,[5],12:i:-, *S.* ser. Typhimurium, and *S.* ser. Enteritidis, as reported by previous studies in the Netherlands [25], Greece [26], and the United States [27]. The serotypes that were frequently identified from urinary tract infections were *S.* ser. Typhimurium 1,4,[5],12:i:-, *S.* ser. Derby, and *S.* ser. Typhimurium, followed by *S.* ser. Enteritidis. This latter serotype together with *S.* ser. Typhimurium were the two serotypes most often associated with urinary tract infections in Brazil [28], Spain [29], and the United States [30], whereas *S.* ser. Derby was sporadically detected in urinary infections in the past [30,31].

Investigation of the susceptibility of *Salmonella* serotypes toward drugs indicated that most strains were resistant to at least one antibiotic and that the highest levels of resistance were against tetracycline, ampicillin, quinolones, and gentamicin. The high levels of resistance we observed is shared by previous reports and is of particular concern, since these antibiotics are commonly used in the first-line treatment of human and animal infections [12]. For instance, fluoroquinolones are the gold standard in treatment against invasive salmonellosis in human medicine, and ampicillin and tetracycline are widely used in veterinary medicine as first-line treatments [32]. The resistance to third-generation cephalosporines we noted was moderate, albeit higher than that reported previously [22,33]. In the present study, chloramphenicol had the lowest level of resistance, likely due to its use in veterinary medicine having been prohibited in Europe since January 1997 [34]. Though antimicrobial resistance to chloramphenicol is still present, it appears to be decreasing [35]. To compare antibiotic resistance patterns, we divided the study into two time periods. Resistance rates significantly decreased during the latter half of the study period, especially for *S.* ser. Typhimurium 1,4,[5],12:i:-, *S*. ser. Typhimurium, *S.* ser. Napoli, *S.* ser. Derby, *S.* ser. Brandenburg, and *S.* ser. Infantis. During both periods, we observed a reduction in resistance to tetracycline, ampicillin, quinolones, and cefotaxime. Similarly, a decline in resistance to tetracyclines and ampicillin in *Salmonella* from humans was observed in other European countries (nine and ten countries, respectively) over the period from 2015 to 2019, which was particularly true for *S.* ser. Typhimurium [12]. Decreasing trends of resistance were more commonly observed for ampicillin in *S.* ser. Typhimurium (nine countries) and for tetracycline in *Salmonella* spp. (eleven countries), *S.* ser. Typhimurium (nine countries), *S.* ser. Typhimurium 1,4,[5],12:i:- (six countries), and *S.* ser. Infantis (two countries). Despite the decline, resistance to these antibiotics remains high in bacteria isolated from humans and animals [12]. These resistance data are of particular concern for clinicians who use antimicrobials (e.g., fluoroquinolones and cephalosporins) in the treatment of children and in the early treatment of severe gastroenteritis or invasive infections in adults.

Worldwide actions have been undertaken to prevent the emergence of drug-resistant bacteria and to promote food safety and public health through plans to ban or reduce the use of certain antimicrobials. The development of AMR can be slowed by restricting inappropriate use of antimicrobials and by improving hygiene conditions and practices in healthcare settings or in the food chain to reduce the transmission of resistant microorganisms, where more than one cause may play a role. Furthermore, the application of European regulations concerning the utilization of antibiotics in veterinary medicine has had a positive impact on slowing the spread of AMR. The JIACRA report also identifies links between antimicrobial consumption in animals and AMR in bacteria from food-producing animals, which is associated with antimicrobial resistance in bacteria from humans. Data also show that the use of antibiotics has decreased and is now lower in food-producing animals than in humans [36].

Regarding MDR strains, our data showed that almost 50% of *Salmonella* strains are resistant to more than three classes of antibiotics. According to European data [9], MDR is highest in *S.* ser. Typhimurium, *S.* ser. Infantis, and *S.* ser. Typhimurium 1,4,[5],12:i:-. In our study, the number of MDR isolates was far higher in the first half than the second half of the study period, in which the significant reduction in the occurrence of *S.* ser. Typhimurium 1,4,[5],12:i:-, *S.* ser. Typhimurium, *S.* ser. Enteritidis, *S.* ser. Napoli, *S.* ser. Derby, and *S*. ser. Brandenburg may be ascribed to the implementation of Italian and European control programs for the eradication of *Salmonella* in poultry [37].

We observed high levels of resistance in *S.* ser. Typhimurium 1,4,[5],12:i:-, *S.* ser. Typhimurium, *S.* ser. Enteritidis, *S.* ser. Infantis, S. ser. Derby, and *S.* ser. Newport against ampicillin and tetracycline. Many human *Salmonella* strains were resistant to ampicillin, sulfonamides, and tetracyclines, as reported in other countries [12].

This study has benefited from the well-structured and coordinated surveillance health system implemented in the region. Collaboration among different health services has made possible the collection of a significant amount of data on human salmonellosis in the long term, enabling the evaluation of temporal trends of *Salmonella* strains and identify the main AMR patterns occurring in human infections. Notwithstanding, our study is limited to only six antibiotic classes because of the monitoring of AMR involving other antimicrobials was sometimes interrupted, thus preventing comparisons over time. Moreover, our survey was restricted to a specific geographical context, not allowing our findings to be generalized to other Italian or European regions.

## 5. Conclusions

Our study focused on the occurrence and the antibiotic resistance of *Salmonella* spp. detected in humans in northwest Italy over a 10 year period. High AMR levels were uncovered among *Salmonella* strains toward tetracycline, ampicillin, and quinolones in particular. These data provide supplementary information about AMR in human strains of *Salmonella*. The decreasing trend of AMR experienced by *Salmonella* in Piedmont is consistent with data from the European Union and demonstrates the effectiveness of measures implemented in human and veterinary medicine.

## Figures and Tables

**Table 1:** Prevalence and 95% confidence interval (CI) of most prevailing *Salmonella enterica* subspp. enterica serotypes identified in human patients from Piedmont during 2012–2021.

Serotype	No. Isolates (*n* = 4814)	Prevalence (95% CI)
Typhimurium 1,4,[5],12:i:-	2188	45.5 (44.0–46.9)
Typhimurium	666	13.8 (12.9–14.8)
Enteritidis	453	9.4 (8.6–10.3)
Napoli	172	3.6 (3.1–4.1)
Derby	164	3.4 (2.9–4.0)
Brandenburg	129	2.7 (2.2–3.2)
Rissen	97	2.0 (1.6–2.5)
Infantis	84	1.7 (1.4–2.2)
London	68	1.4 (1.1–1.8)
Bovismorbificans	44	0.9 (0.7–1.2)
Minor serotypes	749	15.6 (14.5–16.6)

## Data Availability

Not applicable.

## Supplementary Materials

The following supporting information can be downloaded at: https://www.mdpi.com/article/10.3390/pathogens12010089/s1, Table S1: Prevalence of the *Salmonella enterica* serotypes involved in human infections in Piedmont, northwestern Italy, 2012–2021.
