# Supplementary material for: Monitoring of Antimicrobial Resistance of Salmonella Serotypes Isolated from Humans in Northwest Italy, 2012–2021
Source: Pathogens. 2023 Jan 5;12(1):89. doi: 10.3390/pathogens12010089 (PMC9865215; doi:10.3390/pathogens12010089)
Supplement: Supplementary file 1 [file pathogens-12-00089-s001.zip › pathogens-2097466-supplementary.pdf]

Table S1. Prevalence of the *Salmonella enterica* serotypes involved in human infections in Piedmont, northwestern Italy, 2012-2021.

***Salmonella* serotype**	**No. of isolates (*n*= 4814)**	**Prevalence (%)**
4,12:-:-	2	0.04
6,7:-:1,5	1	0.02
6,7:c:1,5	5	0.1
Aba	1	0.02
Abony	1	0.02
Adjame	2	0.04
Afula	8	0.2
Agama	21	0.4
Agbeni	4	0.1
Agona	14	0.3
Ahmadi	1	0.02
Ajiobo	2	0.04
Altona	2	0.04
Amherstiana	1	0.02
Amoutive	1	0.02
Anatum	5	0.1
Assinie	1	0.02
Baildon	2	0.04
Bareilly	2	0.04
Benfica	1	0.02
Blockley	4	0.1
Bongori	1	0.02
Bonn	2	0.04
Borbeck	1	0.02
Bovismorbificans	44	0.9
Bradford	1	0.02
Braenderup	12	0.2
Brandenburg	129	2.7
Bredeney	39	0.8
Budapest	1	0.02
Cannonhill	1	0.02
Carrau	1	0.02
Chester	12	0.2
Chingola	1	0.02
Choleraesuis	1	0.02
Clackamas	1	0.02
Coeln	16	0.3
Colindale	3	0.1
Concord	2	0.04
Coogee	1	0.02
Dabou	1	0.02

Salmonella serotype	No. of isolates (*n*= 4814)	Prevalence (%)
Derby	164	3.4
Dublin	4	0.1
Duisburg	2	0.04
Durham	2	0.04
Eastbourne	1	0.02
Eingedi	3	0.1
Enteritidis	453	9.4
Eppendorf	1	0.02
Eschberg	2	0.04
Euston	3	0.1
Farsta	1	0.02
Fischerhuette	1	0.02
Fyris	2	0.04
Galiema	1	0.02
Gallinarum	1	0.02
Gatow	1	0.02
Give	41	0.9
Goettingen	3	0.1
Goldcoast	43	0.9
Gueuletapee	1	0.02
Hadar	10	0.2
Haifa	1	0.02
Havana	1	0.02
Hessarek	4	0.1
Hofit	1	0.02
Houston	8	0.2
Hvittingfoss	1	0.02
Ibadan	2	0.04
Idikan	1	0.02
Indiana	4	0.1
Infantis	84	1.7
Isangi	2	0.04
Itami	1	0.02
Ituri	1	0.02
Javiana	2	0.04
Johannesburg	1	0.02
Kano	1	0.02
Kapemba	17	0.4
Kedougou	6	0.1
Kentucky	20	0.4
Kenya	7	0.1
Kimuenza	2	0.04
Kisii	1	0.02
Kortrijk	2	0.04
Salmonella **serotype**	**No. of isolates**	**Prevalence**

	**(*n*= 4814)**	**(%)**
Kottbus	13	0.3
Lamphun	1	0.02
Larochelle	1	0.02
Litchfield	11	0.2
Livingstone	9	0.2
Lomalinda	3	0.1
Lomita	1	0.02
London	68	1.4
Madelia	1	0.02
Manhattan	2	0.04
Matopeni	2	0.04
Mbandaka	2	0.04
Mikawasima	3	0.1
Mkamba	1	0.02
Montevideo	6	0.1
Muenchen	22	0.5
Muenster	4	0.1
Namibia	1	0.02
Nanga	1	0.02
Napoli	172	3.6
Nchanga	1	0.02
Ndolo	1	0.02
Newport	24	0.5
Nigeria	4	0.1
Nohanga	1	0.02
Oakland	1	0.02
Ohio	1	0.02
Oranienburg	1	0.02
Orion	3	0.1
Oritamerin	2	0.04
Othmarschen	1	0.02
Pakistan	1	0.02
Panama	18	0.4
Paratyphi A	2	0.04
Paratyphi B	2	0.04
Paratyphi C	3	0.1
Pomona	1	0.02
Poona	3	0.1
Potsdam	1	0.02
Reading	1	0.02
Rissen	97	2.0
S. enterica subsp. arizonae (IIIa)	1	0.02
S. enterica subsp. diarizonae (IIIb)	6	0.1
S. enterica subsp. houtenae (IV)	2	0.04
***Salmonella* serotype**	**No. of isolates (*n*= 4814)**	**Prevalence (%)**

S.43:Z4,Z23:-	1	0.02
S. enterica subsp. salamae (II)	30	0.6
Saintpaul	11	0.2
Sandiego	2	0.04
Schleissheim	2	0.04
Schwarzengrund	2	0.04
Senftenberg	3	0.1
Seremban	2	0.04
Singapore	1	0.02
Sinstorf	1	0.02
Stanley	10	0.2
Strathcona	10	0.2
Szentes	2	0.04
Takoradi	1	0.02
Tamale	1	0.02
Telelkebir	1	0.02
Tennessee	1	0.02
Thompson	40	0.8
Trachau	2	0.04
Travis	1	0.02
Tsevie	3	0.1
Tshiongwe	1	0.02
Typhi	11	0.2
Typhimurium	666	13.8
Typhimurium 1,4,[5],12:I:-	2188	45.5
Uganda	2	0.04
Umbilo	2	0.04
Vejle	2	0.04
Veneziana	27	0.6
Virchow	13	0.3
Wangata	1	0.02
Weltevreden	2	0.04
Weston	1	0.02
Winston	1	0.02
Yellowknife	1	0.02
York	1	0.02
Zaiman	3	0.1
Zanzibar	1	0.02
Zwickau	1	0.02
